# Type 2 diabetes among people with psychosis in Finland – prevalence, treatment balance, and mortality

**DOI:** 10.1192/j.eurpsy.2026.12162

**Published:** 2026-07-31

**Authors:** J. Keinänen, M. Holm, K. Suokas, J. Haapakoski, A. Tahkola, T. Kauppala, E. Liukko, L. Schildt, S. Vanhamäki, S. Metso, J. Suvisaari

**Affiliations:** 1 National Institute for Health and Welfare; 2 Psychiatry, Helsinki University Hospital; 3 University of Helsinki, Helsinki, Finland

## Abstract

**Introduction:**

Individuals with psychosis experience markedly reduced life expectancy and a high burden of cardiometabolic comorbidities. Multiple risk factors for type 2 diabetes (T2D) accumulate in people with psychosis. Despite this high burden, real-world data on T2D management and outcomes among people with psychosis remain limited, underscoring the need for integrated approaches that address both psychiatric and metabolic health.

**Objectives:**

Aim of the study was to compare baseline cardiometabolic profiles, treatment balance and mortality between those with psychosis and T2D and those with T2D alone.

**Methods:**

We identified individuals younger than 60 years with type 2 diabetes (T2D) from the Finnish diabetes register who were also recorded in the psychosis care quality register. The diabetes register includes all Finnish residents who were alive and had a documented diabetes diagnosis in or after 2018. We compared T2D treatment balance and all-cause mortality between those who developed T2D after a prior diagnosis of a psychotic disorder and those with T2D only, with follow-up through the end of 2023.

**Results:**

Table 1 shows the baseline characteristics of the study population. Overall, the psychosis plus diabetes group demonstrated a more favorable cardiometabolic profile at baseline. Individuals with a history of psychosis prior to diabetes diagnosis had a significantly increased risk of mortality compared to individuals without prior psychosis (Table 2, Figure 1). In the Cox proportional hazards model, prior psychosis was associated with more than a two-fold increase in the hazard of death (HR = 2.08, 95% CI 1.90–2.27, p < 0.001). Older age and high clinical risk status were also associated with elevated mortality risk, whereas female sex was associated with lower mortality.

**Table 1.** Baseline characteristics of the study population Table 1. Long description.

Variable	Psychosis + T2D (n=8034)	T2D (n=134844)	All (n=142878)	p-value
**Age (mean (SD))**	46.36 (9.50)	48.82 (8.50)	48.68 (8.58)	<0.001
**Gender Female (%)**	3658 (45.5)	54932 (40.7)	58590 (41.0)	<0.001
**HbA1c**	47.97 (12.90)	51.56 (13.20)	51.36 (13.21)	<0.001
**Low-density lipoprotein cholesterol**	2.57 (0.78)	2.67 (0.82)	2.66 (0.82)	<0.001
**Glomerular filtration rate**	96.19 (17.74)	95.36 (16.52)	95.41 (16.59)	<0.001

**Table 2.** Adjusted Cox regression results for all-cause mortality Table 2. Long description.

Predictor	Hazard Ratio	95% CI	p-value
Psychosis before diabetes vs only T2D	**2.08**	1.90 – 2.27	<0.001
Age (per year increase)	1.03	1.02 – 1.03	<0.001
Female sex (vs male)	0.65	0.61 – 0.69	<0.001
High medical risk (vs low)	2.66	2.51 – 2.81	<0.001

**Image:**
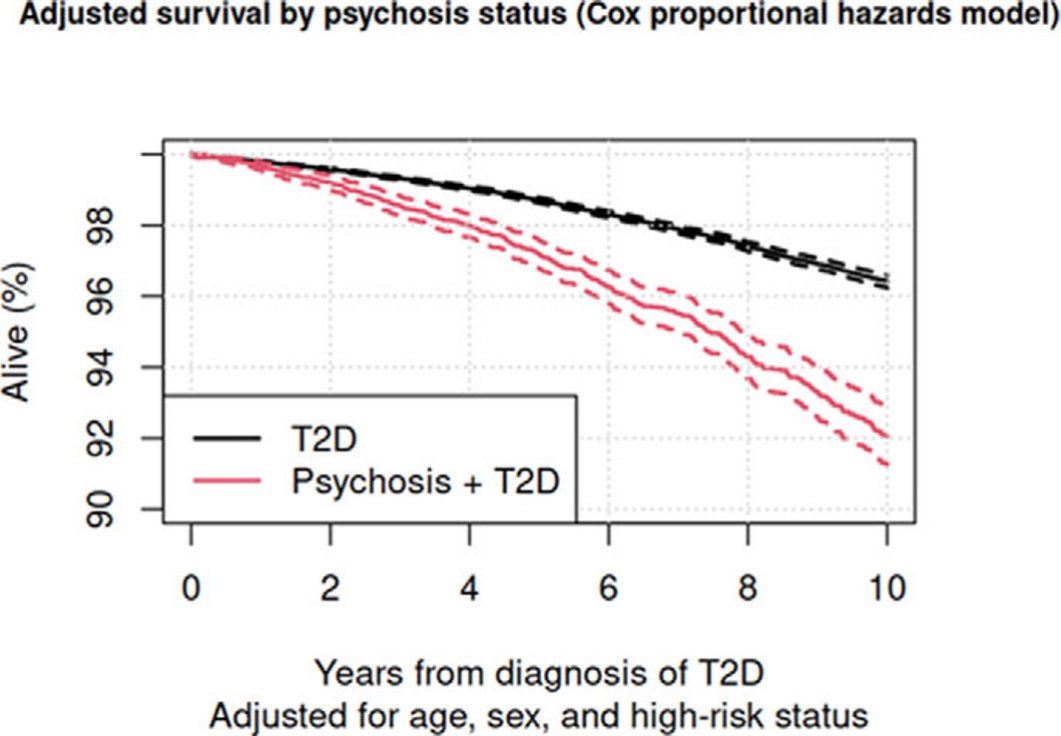
Image: Long description.

**Conclusions:**

In this nationwide cohort, individuals with psychosis and type 2 diabetes exhibited lower HbA1c and LDL levels at baseline compared with those with T2D alone, suggesting better glycemic and lipid treatment balance in the psychosis group. Despite the superior cardiometabolic markers, prior psychosis was associated with a increased risk of all-cause mortality.

**Disclosure of Interest:**

None Declared

## Long descriptions

### Table 1. Long description

The table consists of five columns: Variable, Psychosis plus T 2 D (n equals 8034), T 2 D (n equals 134844), All (n equals 142878), and p-value.

* Age (mean (S D)): Psychosis plus T 2 D is 46.36 (9.50), T 2 D is 48.82 (8.50), All is 48.68 (8.58), p-value is less than 0.001.

* Gender Female (percentage): Psychosis plus T 2 D is 3658 (45.5), T 2 D is 54932 (40.7), All is 58590 (41.0), p-value is less than 0.001.

* H b A 1 c: Psychosis plus T 2 D is 47.97 (12.90), T 2 D is 51.56 (13.20), All is 51.36 (13.21), p-value is less than 0.001.

* Low-density lipoprotein cholesterol: Psychosis plus T 2 D is 2.57 (0.78), T 2 D is 2.67 (0.82), All is 2.66 (0.82), p-value is less than 0.001.

* Glomerular filtration rate: Psychosis plus T 2 D is 96.19 (17.74), T 2 D is 95.36 (16.52), All is 95.41 (16.59), p-value is less than 0.001.

Navigate back to Table 1..

### Table 2. Long description

The table consists of four columns: Predictor, Hazard Ratio, 95 percent C I, and p-value.

* Psychosis before diabetes versus only T 2 D: Hazard Ratio 2.08, 95 percent C I 1.90 to 2.27, p-value less than 0.001.

* Age per year increase: Hazard Ratio 1.03, 95 percent C I 1.02 to 1.03, p-value less than 0.001.

* Female sex versus male: Hazard Ratio 0.65, 95 percent C I 0.61 to 0.69, p-value less than 0.001.

* High medical risk versus low: Hazard Ratio 2.66, 95 percent C I 2.51 to 2.81, p-value less than 0.001.

Navigate back to Table 2..

### Image: Long description

The X-axis represents Years from diagnosis of T 2 D, ranging from 0 to 10 in increments of 2. The Y-axis represents the percentage of individuals Alive (%), ranging from 90 to 100 in increments of 2.

Two main survival curves are plotted, each accompanied by dashed lines representing confidence intervals.

* The T 2 D group (black line) shows a gradual, nearly linear decline in survival, starting at 100% at year 0 and ending at approximately 96.5% at year 10.

* The Psychosis plus T 2 D group (red line) shows a significantly steeper decline. While it also starts at 100% at year 0, it drops more rapidly after year 2, reaching approximately 92% survival by year 10.

The gap between the two groups widens progressively over the 10-year period, indicating a higher hazard for the psychosis group. The graph is noted as being adjusted for age, sex, and high-risk status.

Navigate back to Image:.

